# Upregulation of β2-microglobulin expression in progressive human oral squamous cell carcinoma

**DOI:** 10.3892/or.2011.1613

**Published:** 2011-12-30

**Authors:** QIAN JIANG, SDEK PATIMA, DONG-XIA YE, HONG-YA PAN, PIN ZHANG, ZHI-YUAN ZHANG

**Affiliations:** 1 Department of Oral and Maxillofacial Surgery, Ninth People's Hospital, Shanghai Research Institute of Stomatology, Shanghai Key Laboratory of Stomatology, Shanghai, P.R. China; 2 Division of Cardiology, University of California at Los Angeles, The Cardiovascular Research Laboratory, Department of Medicine, David Geffen School of Medicine, Los Angeles, CA, USA

**Keywords:** oral squamous cell carcinoma, β2-microglobulin, invasion, metastasis, immunohistochemical staining

## Abstract

The aim of the present study was to investigate β2-microglobulin (β2-M) expression in normal oral mucosa and progressive oral squamous cell carcinoma (OSCC) and to assess the clinical significance of β2-microglobulin expression. The study included 10 cases of normal oral mucosa epithelium specimens, 55 cases of primary OSCC specimens, and 25 cases of OSCC metastasis specimens. Immunohistochemistry was used to determine β2-M expression, and its correlation with clinicopathological factors in progressive OSCC was evaluated. Immunohistochemistry showed that strong β2-M expression was significantly asscociated with tumor size (T3, T4 vs. T1, T2; P=0.001), positive node status (N positive vs. N negative; P=0.000) and advanced clinical stage (III, IV vs. I, II, P=0.000) in primary OSCC lesions. Compared to primary OSCC lesions, the frequency of β2-M expression was significantly increased in metastatic OSCC lesions (P=0.02). In addition, *in vitro* results from Western blotting showed increased β2-M expression in the two OSCC lines studied. Therefore, we speculate that the up-regulation of β2-M expression may contribute to the oncogenesis of human oral mucosa, tumor invasion and metastasis.

## Introduction

Oral squamous cell carcinoma (OSCC) is the sixth most common cancer in the world (1). Postoperative quality of life for patients with OSCC has improved in recent years (2). However, the 5-year survival rate has not improved significantly. Furthermore, 30–40% of patients without evidence of nodal disease at resection eventually die from metastatic spread (3). The identification of biomarkers for evaluating the progression of OSCC is therefore urgent.

It has been suggested that β2-microglobulin (β2-M) expression in tissues may be involved in OSCC progression and metastasis (4). β2-M is a non-glycosylated protein with a molecular mass of 11,800 Da and is synthesized by all nucleated cells (5). It is present on the surface of all nucleated cells except for red blood cells (6). β2-M forms the β chain of the major histocompatibility complex (MHC) class I molecule [also known as human leukocyte antigens (HLAs) in humans] and has a 7-stranded β-pleated structure, which is believed to function in antigen presentation to cytotoxic (CD8^+^) T lymphocytes (7). Upon recognition of foreign peptide antigens on cell surfaces, T cells actively bind and lyse antigen-presenting cancer cells. In β2-M-deficient mice, antibody (Ab) responses are defective, and natural killer (NK) cells with increased sensitivity attack cells lacking the MHC class I molecule (8,9). In addition to the roles in immunity, the level of β2-M is associated with proliferation, apoptosis and metastasis in several cancer types (10,11), and is a predictor of survival in patients with certain types of cancer (12). β2-M was found to promote the growth of human renal cell carcinoma through the activation of the protein kinase A, cyclic AMP-responsive element-binding protein, and vascular endothelial growth factor axis (11). Overexpression of β2-M in human prostate cancer cell lines leads to inhibition of tumor growth *in vivo* and using the β2-M Ab to interrupt β2-M signaling in human prostate cancer cell lines inhibits cancer cell growth and induces cell apoptosis (13).

The aim of this study was to investigate β2-M expression in normal oral mucosa and progressive OSCC and to assess the clinical significance of β2-M expression. The results of our study may contribute to a better understanding of the clinical significance of alterations in β2-M expression and may lead to further insights into the mechanisms to control progression and metastatic spread of tumor cells in OSCC patients.

## Materials and methods

### Cell cultures

Normal human oral keratinocytes (NHOKs) and human immortalized oral epithelial cells (HIOECs) (14,15) were cultured in defined keratinocyte medium-SFM (cat. no. 10744; Gibco, USA). CAL27 was purchased from ATCC (Manassas, VA). The OSC-4 cells were from Kochi Medical School, Japan. The CAL27 cells were cultured in DMEM (Invitrogen) with supplements (10% fetal bovine serum, 1% glutamine and 1% penicillin-streptomycin). The OSC-4 cells were cultured in RPMI-1640 (Invitrogen) with the same supplements.

### Western blotting

Protein extracts were prepared from 1×10^6^ cells using standard procedures. Cell lysates containing 20 μg protein were subjected to Western blot analysis. The primary Ab was monoclonal mouse anti-β2-M (sc-13565, 1:1000; Santa Cruz Biotechnology Inc.), and tubulin was detected as input control using monoclonal mouse anti-tubulin (T9026, 1:50,000; Sigma), Blots were developed with Immobilon Western Chemiluminescent HRP Substrate (Millipore, USA).

### Tissue specimens

Tissue specimens were obtained from the files of the Department of Oral and Maxillofacial Surgery, Shanghai Ninth People's Hospital, Shanghai Jiao Tong University School of Medicine, China. All tissue samples had been fixed in 10% buffered formalin and embedded in paraffin wax. For primary OSCC lesions obtained from 50 untreated patients, who underwent surgery between 2008 and 2009, clinicopathological data, including gender, age, tumor site, primary tumor stage (T), lymph node status (N) and tumor-node-metastasis (M) were obtained from the patient clinical records and pathological reports (Table I). Clinical stage was determined according to the 2002 American Joint Committee on Cancer (AJCC) staging system. Histopathological diagnosis and grading were confirmed using haematoxylin and eosin-stained sections according to the criteria mentioned in ‘Histological Typing of Tumors of the Upper Respiratory Tract and Ear’, WHO, 2nd edition. All data were re-examined independently by two of the authors. Metastatic OSCC lesions from 25 patients were obtained prior to biotherapy or chemotherapy between 2008 and 2009, and data including gender, age, and metastatic type was collected. (Table II). Analyses of the tissue samples are documented in Tables III-V. Histologically normal oral mucosa samples were obtained from 10 patients who underwent dental extractions. The human studies were approved by the institutional ethics committee.

### Immunohistochemical staining

Formalin-fixed, paraffin-embedded tissue sections were dewaxed with xylene and rehydrated by passage through decreasing concentrations of ethanol (100–80%). Endogenous peroxidase activity was blocked by a 20-min incubation at room temperature with 3% H_2_O. The sections were heated using a water bath at 100°C with 0.01 M citrate buffer solution (pH, 6.0) for 20 min, and incubated with an optimal amount of affinity-purified monoclonal mouse anti-human β2-M (sc-13565, 1:50; Santa Cruz Biotechnology) overnight at 4°C. Sections were stained with liquid DAB substrate-chromogen, and counterstained with hematoxylin. Negative controls were carried out by omitting the primary Ab. The percentage of stained tumor cells in each lesion was enumerated independently by two investigators who had no knowledge of the patient characteristics. Variations in the percentage of stained cells as counted were within a 10% range. We scored the staining results according the report of Kageshita *et al* (16). Briefly, OSCC lesions consisting of >75% immunostained OSCC cells within the entire lesion were scored as homogeneously positive, those having 25–75% immunostained OSCC cells were heterogeneously positive, and those with <25% immunostained OSCC cells were negative.

### Statistical analysis

Several clinicopathological factors were evaluated in the primary OSCC lesions, including gender, age (≤61 years vs. >61 years), T stage (T1, T2 vs. T3, T4), N status (negative vs. positive) and clinical stage (stage I, II vs. stage III, IV). Pearson Chi-square test, Continuity Correction test and Fisher's exact test were used to evaluate the correlation between the clinicopathological variables and the β2-M staining score using SPSS software v13.0 (SPSS Inc., USA). Differences in the β2-M staining score between primary OSCC samples and metastatic OSCC samples were also analyzed using the Chi-square test. A P-value <0.05 was considered to denote significant difference.

## Results

### Expression of β2-M in primary cultured NHOKs and HIOECs and the OSCC cell lines

**Figure 1:**
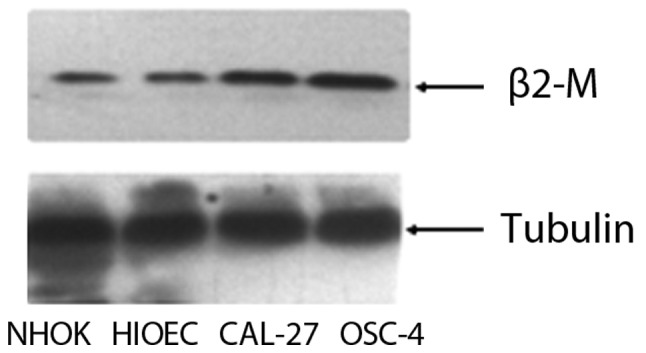
β2-M protein is up-regulated in oral squamous cell carcinoma. Western blot analysis of β2-M protein expression in NHOKs, HIOECs, CAL-27 cells and OSC-4 cells. The β2-M protein expression in CAL-27 and OSC-4 cells was higher than that in the NHOKs and HIOECs. Data are representative of 3 independent experiments.

We compared the expression levels of β2-M in NHOKs and HIOECs and in the two OSCC cancer cell lines (OSC and CAL27) by Western blotting. NHOKs were isolated and cultured as described (14). HIOECs were established by overexpression of HPV16 E6 and E7 protein (14). Western blot analysis revealed that β2-M protein expression was increased in the OSC and CAL27 cells compared to the NHOKs and HIOECs (Fig. 1).

### Expression of β2-M in normal oral mucosa epithelial and OSCC tissue specimens

**Figure 2:**
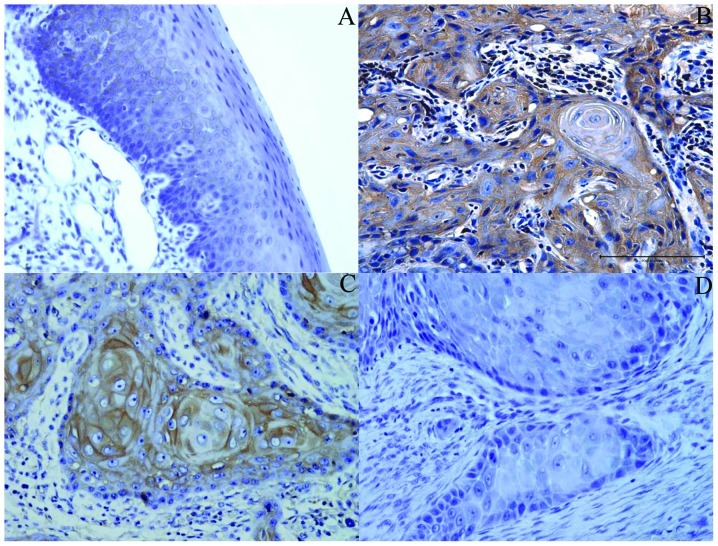
(A) Immunohistochemical staining of β2-M in normal oral mucosa tissue and (B-D) OSCC tissue. (A) Faint plasma membrane staining was observed in normal oral mucosa epithelial cells. Stromal cells such as fibroblasts and fibrocytes were not stained. β2-M staining was classified according to three scales in OSCC: (B) homogeneously positive, (C) heterogeneously positive and (D) negative. (A-D, magnification ×400).

We performed immunohistochemical staining using normal oral mucosa and OSCC tissue specimens. Ten human normal oral mucosa samples and 75 human OSCC lesions (50 primary OSCC and 25 metastatic OSCC samples) were included. In the human normal oral mucosa, a faint but consistent staining was observed, mainly in the plasma membrane in oral mucosa epithelial cells. Stromal cells such as fibroblasts and fibrocytes were not stained by the anti-β2-M Ab (Fig. 2A). Most of the OSCC (88%) tissue sections showed distinct homogeneous (Fig. 2B) or heterogeneous staining (Fig. 2C), mainly in the cytoplasm and cytoplasmic membrane of tumor epithelial cells. However, in a few primary OSCC tissues, no staining or staining with weak intensity for β2-M was noted in the cytoplasm and cytoplasmic membrane of tumor epithelial cells (Fig. 2D). Compared with normal oral mucosa specimens, the frequency of β2-M expression was significantly increased in OSCC (P=0.031) (Table III).

### Association of β2-M expression with various clinicopathological features in primary OSCC tissues

Of the 50 primary OSCC samples, 26 (52%) exhibited a homogeneous distribution of β2-M staining, and 15 (30%) exhibited a heterogeneous distribution within the OSCC cells, while 9 (18%) were negative for β2-M staining (Table IV). Of the 23 patients classified as T1, T2 in 50 primary OSCC cases, 9 (39.1%) showed heterogenous staining and 8 (34.8%) showed negative staining, while only 6 (26.1%) exhibited homogeneous staining. In contrast, of the 27 patients classified as T3, T4, 20 (74.1%) presented with homogeneous staining, whereas only 6 (22.2%) showed heterogenous staining and 1 (3.7%) showed negative staining. Compared with primary OSCC of T1, T2 stage, the intensity of β2-M expression was significantly increased in the primary OSCC specimens of T3, T4. Up-regulation of β2-M expression was also associated with lymph node invasion of OSCC. β2-M expression was significantly increased in N-positive patients compared to N-negative patients (82.8 vs. 9.5%, P=0.000). Regarding clinical stage, in highly malignant stages (III, IV) 67.6% of samples showed homogeneous staining whereas in low malignant stages (I, II), only 1% of samples showed homogeneous staining. These data suggest that the staining scores for β2-M were significantly associated with large tumor size (T3, T4 vs. T1, T2, P=0.001), positive nodal status (N-positive vs. N-negative, P=0.000), and advanced clinic stage (III, IV vs. I, II, P=0.000) (Table IV). In contrast, there were no correlations between β2-M expression and gender, age, smoking and alcohol consumption, and pathologic grade.

### Up-regulation of β2-M expression in OSCC tissues correlates with tumor metastasis

**Figure 3:**
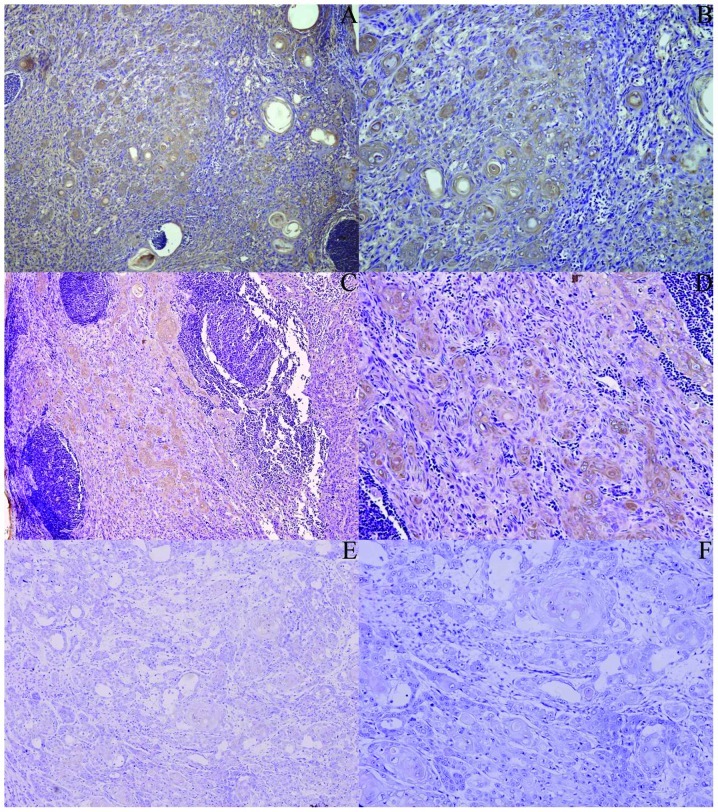
β2-M immunohistochemistry of metastatic oral squamous cell carcinoma tissues. (A and B) Homogeneously positive; (C and D) heterogeneously positive. (E and F) Negative controls performed by substitution of the primary antibody with mouse non-immune IgG. (A, C, E: magnification ×100; B, D, F: magnification ×200).

Intensity of β2-M staining in OSCC metastatic lesions was significantly stronger than that in the primary OSCC lesions (Fig. 3). Ninenty-two percent of metastatic lesions exhibited a homogeneous distribution of β2-M expression while 52% of primary OSCC lesions exhibited homogeneous staining for β2-M (P=0.027) (Table V).

## Discussion

In the present study, β2-M expression in OSCC lesions was evaluated and correlated with tumor progression and metastasis in OSCC patients. The results showed that β2-M expression was up-regulated in OSCC cell lines and OSCC lesions, and was associated with OSCC progression, invasion and metastasis. Consistent with our results, it was previously found that the suppression of β2-M expression using small interfering RNA (siRNA) was sufficient to decrease cell migration and invasion *in vitro* (4). The results of our and other research studies (4), indicate that OSCC lesions should be included in the spectrum of tumors with increased levels of β2-M expression.

Recent studies have used a wide range of experimental approaches to assess the mitogenic role of β2-M in malignancies. (17–19). These studies have provided strong evidence to show that β2-M acts similarly to a prototypical oncogenic factor capable of stimulating growth and progression of various types of cancers, including breast cancer (17), prostate cancer (18), lung cancer (19), gastrointestinal (20), nasopharyngeal cancers (21), multiple myeloma (22), and particularly, lymphocytic malignancies (23), such as non-Hodgkin's lymphoma and multiple myeloma. Similar studies have also reported that β2-M is a growth-promoting factor contributing to the growth and progression of renal cell carcinoma (24,25).

However, previous studies have shown that β2-M/MHC class I can serve as important signal-transducing molecules in regulating tumor immunity and progression (26). Increased susceptibility to tumor formation was noted in β2-M gene-knockout mice, which suggests potential regulation of cancer growth by β2-M (26). Loss of β2-M expression is clinically important as it has been described in various patient-derived tumor cells, such as in melanomas (27) and cervical carcinoma (28). The possible explanation is that β2-M is expressed at a constant level on the cell surface. When expression of the β2-M molecule is below a normal level, defects in the β2-M/MHC class I signaling pathway may result in tumor immune escape. When expression of the β2-M molecule is higher than normal, β2-M promotes tumorigenesis and metastasis as an oncogene.

In some cancers, immunohistochemical evidence suggests that absence of functional (β2-M-associated) HLA class I molecules may be due to a mutational loss of β2-M (29); and in other cancers, a decreased level or the loss of β2-M in tumor cells was found due to the loss of the β2-M locus, or promoter methylation (30,31). Under these conditions, loss of β2-M prevents the synthesis of wild-type β2-M protein, which may lead to alterations in MHC class I surface expression. In our study, was not observed loss of β2-M in the OSCC lesions. In contrast, levels of β2-M expression were up-regulated in progressive OSCC lesions. The balance of β2-M expression at the cell surface was disturbal, which contributed to human cancer growth. Therefore, β2-M has a wider function than just a housekeeping gene or the role on stabilization and presentation of MHC class I molecular in cells.

Recently, marked antitumor activity has been observed by down-regulation of β2-M levels using either sequence-specific siRNA or antibodies in cases of both solid tumors and blood malignancies (13). In prostate cancer and renal cancer, growth inhibition of tumors was observed when patients were treated with anti-β2-M polyclonal or monoclonal antibodies (32), and in myeloma and other hematological malignancies, tumor cell apopotosis was observed using monoclonal β2-M Ab and sequence-specific siRNA to β2-M (33). Thus, β2-M, as an oncogenic factor in various cancer types, appears to be an excellent new target for interrupting human cancer growth. In our study, the association of β2-M expression with progression and metastasis of OSCC lesions was statistically significant. Whether we can inhibit OSCC progression, invasion or migration by using a similar anti-β2-M polyclonal or monoclonal antibody needs further study.

## Figures and Tables

**Table I:** Profiles of the patients with primary oral squamous cell carcinoma.

No	Gender	Age (years)	Location stage	Pathological grade	TNM	Clinical stage	Smoking Alcohol consumption	β2-microglobulin
1	M	54	Palate	G1	T3N1M0	III	Yes	Homogeneous
2	F	63	Gingivae	G2	T3N1M0	III	No	Homogeneous
3	M	48	Tongue	G2	T2N1M0	III	Yes	Homogeneous
4	F	54	Tongue	G2	T2N1M0	III	No	Homogeneous
5	F	62	Floor of the mouth	G1	T4N1M0	IV	Yes	Homogeneous
6	M	35	Tongue	G2	T4N1M0	IV	No	Homogeneous
7	F	71	Tongue	G3	T3N1M0	III	No	Homogeneous
8	M	55	Tongue	G2	T4N1M0	IV	No	Homogeneous
9	F	64	Gingivae	G1	T3N0M0	III	Yes	Heterogeneous
10	M	65	Tongue	G2	T2N0M0	II	No	Negative
11	M	64	Tongue	G2	T3N0M0	III	Yes	Heterogeneous
12	F	54	Tongue	G2	T2N0M0	II	No	Negative
13	F	53	Tongue	G1	T1N0M0	I	No	Negative
14	M	57	Tongue	G2	T2N1M0	III	Yes	Heterogeneous
15	M	40	Buccal	G2	T3N1M0	III	No	Homogeneous
16	M	51	Tongue	G2	T4N2M0	IV	No	Homogeneous
17	M	44	Tongue	G2	T4N1M0	IV	No	Homogeneous
18	F	67	Gingivae	G1	T3N1M0	III	Yes	Homogeneous
19	F	73	Floor of the mouth	G2	T4N1M0	IV	Yes	Homogeneous
20	M	63	Tongue	G2	T2N1M0	III	No	Homogeneous
21	M	58	Tongue	G2	T2N1M0	III	Yes	Homogeneous
22	F	65	Gingivae	G1	T3N1M0	III	Yes	Homogeneous
23	M	58	Buccal	G2	T4N1M0	IV	No	Homogeneous
24	F	53	Palate	G2	T2N1M0	III	No	Heterogeneous
25	M	54	Floor of the mouth	G3	T4N0M0	IV	No	Heterogeneous
26	M	59	Tongue	G1	T1N1M0	III	Yes	Heterogeneous
27	M	60	Tongue	G2	T2N0M0	II	No	Negative
28	F	50	Tongue	G1	T4N0M0	IV	No	Homogeneous
29	M	51	Buccal	G1	T2N0M0	II	No	Heterogeneous
30	M	42	Palate	G3	T2N0M0	II	Yes	Heterogeneous
31	M	65	Tongue	G2	T2N0M0	II	No	Negative
32	M	76	Tongue	G2	T3N0M0	III	Yes	Heterogeneous
33	F	34	Tongue	G2	T2N0M0	II	Yes	Negative
34	F	44	Tongue	G1	T1N0M0	I	Yes	Negative
35	F	68	Buccal	G2	T2N1M0	III	Yes	Heterogeneous
36	M	60	Buccal	G2	T3N1M0	III	No	Homogeneous
37	M	68	Tongue	G2	T4N2M0	IV	No	Homogeneous
38	M	61	Gingivae	G2	T4N1M0	IV	No	Homogeneous
39	F	66	Gingivae	G2	T3N0M0	III	Yes	Negative
40	F	65	Buccal	G2	T4N1M0	IV	Yes	Homogeneous
41	M	65	Tongue	G2	T2N1M0	III	No	Homogeneous
42	M	58	Tongue	G2	T2N0M0	II	Yes	Homogeneous
43	F	72	Gingivae	G2	T3N1M0	III	Yes	Homogeneous
44	M	74	Buccal	G3	T4N1M0	IV	Yes	Homogeneous
45	M	60	Palate	G2	T2N0M0	II	No	Heterogeneous
46	F	73	Tongue	G3	T4N0M0	IV	No	Heterogeneous
47	M	49	Tongue	G2	T1N1M0	III	Yes	Heterogeneous
48	M	54	Tongue	G2	T2N0M0	II	Yes	Negative
49	F	67	Buccal	G3	T4N0M0	IV	No	Heterogeneous
50	F	52	Tongue	G1	T2N0M0	II	No	Heterogeneous

**Table II:** Profiles of the patients with metastatic oral squamous cell carcinoma.

No	Gender	Age (years)	Location	Type	β2-microglobulin
1	M	58	Tongue	Lymph node	Homogeneous
2	M	64	Buccal	Lymph node	Heterogeneous
3	F	65	Tongue	Lymph node	Homogeneous
4	F	67	Tongue	Lymph node	Homogeneous
5	F	67	Tongue	Lymph node	Homogeneous
6	M	56	Tongue	Lymph node	Homogeneous
7	M	54	Tongue	Lymph node	Homogeneous
8	F	60	Tongue	Lymph node	Homogeneous
9	M	61	Tongue	Lymph node	Homogeneous
10	F	80	Buccal	Lymph node	Homogeneous
11	F	81	Buccal	Lymph node	Homogeneous
12	F	86	Tongue	Lymph node	Homogeneous
13	M	88	Tongue	Lymph node	Homogeneous
14	F	49	Buccal	Lymph node	Heterogeneous
15	F	49	Buccal	Lymph node	Homogeneous
16	M	43	Tongue	Lymph node	Homogeneous
17	M	67	Tongue	Lymph node	Homogeneous
18	M	78	Tongue	Lymph node	Homogeneous
19	M	86	Buccal	Lymph node	Homogeneous
20	M	38	Tongue	Lymph node	Homogeneous
21	M	67	Buccal	Lymph node	Homogeneous
22	M	88	Tongue	Lymph node	Homogeneous
23	F	55	Tongue	Lymph node	Homogeneous
24	M	49	Tongue	Lymph node	Homogeneous
25	M	67	Tongue	Lymph node	Homogeneous

**Table III:** β2-microglobulin antigen expression in normal oral mucosa epithelial and oral squamous cell carcinoma specimens.

Staining pattern	Normal oral mucosa epithelial specimens n (%)	Oral squamous cell carcinoma specimens n (%)
Homogeneous	0 (0)	46 (61.3)
Heterogeneous	10 (100)	20 (26.7)
Negative	0 (0)	9 (12.0)
Total	10 (100)	75 (100.0)

P=0.031.

**Table IV:** Association of β2-microglobulin antigen expression with clinicopathological characteristics in primary OSCC lesions.

		β2-microglobulin staining pattern			
			
	N	Homogeneous n (%)	Heterogeneous n (%)	Negative n (%)	P-value
Gender					
Female	19	10 (52.6)	4 (21.1)	5 (26.3)	0.368
Male	31	16 (51.6)	11 (35.5)	4 (12.9)	
Age (years)					
≤61	29	14 (48.3)	9 (31.0)	6 (20.7)	0.748
>61	21	12 (57.1)	6 (28.6)	3 (14.3)	
Smoking and alcohol consumption					
No	28	16 (57.1)	7 (25.0)	5 (17.9)	0.652
Yes	22	10 (45.5)	8 (36.4)	4 (18.2)	
Tumor size					
≤4 cm (T1+T2)	23	6 (26.1)	9 (39.1)	8 (34.8)	**0.001**
>4 cm (T3+T4)	27	20 (74.1)	6 (22.2)	1 (3.7)	
Lymph nodes					
Negative (0)	21	2 (9.5)	10 (47.6)	9 (42.9)	**0.000**
Positive (1–2)	29	24 (82.8)	5 (17.2)	0 (0.0)	
Clinical stage					
Early (I, II)	13	1 (7.7)	4 (30.8)	8 (61.5)	**0.000**
Advanced (III, IV)	37	25 (67.6)	11 (29.7)	1 (2.7)	
Pathological grade					
G1	11	5 (45.5)	4 (36.4)	2 (18.2)	
G2	33	19 (57.6)	7 (21.2)	7 (21.2)	0.237
G3	3	2 (33.3)	4 (66.7)	0 (0.0)	

The percentage of immunoreactive cells in the entire lesion was evaluated microscopically and scored according to the method described by Kageshita *et al* (16): homogeneous, >75% cells stained in the entire lesion; heterogeneous, 25–75% cells stained; negative, <25% cells stained.

**Table V:** Profile of β2-microglobulin antigen expression in primary oral squamous cell carcinoma and metastases.

	Primary OSCC	Metastases
		
Staining pattern	n (%)	n (%)
Homogeneous	26 (52)	20 (80)
Heterogeneous	15 (30)	5 (20)
Negative	9 (18)	0 (0)
Total	50 (100)	25 (100)

P=0.027

## Abbreviations

OSCC: oral squamous cell carcinoma
MHC: major histocompatibility complex
HLAs: human leukocyte antigens
NK: natural killer
HIOECs: human immortalized oral epithelial cells
